# Prevalence of genital chlamydia infection in urban women of reproductive age, Nairobi, Kenya

**DOI:** 10.1186/1756-0500-6-44

**Published:** 2013-02-04

**Authors:** Ruchika Kohli, Walter P Konya, Timona Obura, William Stones, Gunturu Revathi

**Affiliations:** 1 Department of Pathology, Aga Khan University Hospital, P O Box 30270-00100, Nairobi, Kenya; 2 Department of Obstetrics and Gynaecology, Aga Khan University Hospital, Nairobi, Kenya

**Keywords:** Prevalence, Chlamydia, Reproductive age, Vaginal swab, Asymptomatic

## Abstract

**Background:**

*Chlamydia trachomatis* is one of the major causes of sexually transmitted infections throughout the world. Most infections are asymptomatic and remain undetected. Burden of disease in the Kenyan population is not well characterised. This study was done to define the prevalence of genital Chlamydia infection in a representative female population.

**Findings:**

A cross-sectional study design was employed. All women attending out-patient clinics (antenatal, gynaecology, family planning) and accident and emergency departments at two study sites over a five month period were invited to consent to completion of a questionnaire and vaginal swab collection. A rapid point-of-care immunoassay based test was performed on the swabs. Women who tested positive for Chlamydia were offered treatment, together with their partner(s), and advised to come for a follow-up test.

A total of 300 women were tested. The prevalence of genital *Chlamydia trachomatis* was found to be 6% (95% CI 3.31% – 8.69%). The prevalence was higher in women who represented a higher socioeconomic level, but this difference was not significant (p=0.061). Use of vaginal swabs was observed to be a more acceptable form of sample collection.

**Conclusion:**

The prevalence of genital Chlamydia is significant in our female population. There is a justifiable need to institute opportunistic screening programs to reduce the burden of this disease. Rapid and low cost point-of-care testing as a potential component of sexually transmitted infection (STI) screening can be utilised.

## Findings

### Introduction

In Kenya like in many African countries, the syndromic approach is used in the treatment of genital infections. However, this may not be entirely effective for *Chlamydia trachomatis* due to the asymptomatic nature of this infection in many women and the possibility of missing selected infections. Failure to detect infection can have severe consequences including ectopic pregnancy, infertility and pelvic inflammatory disease caused by fibrosis and scarring due to the repair of tissue damaged by Chlamydia induced inflammation [1].

With newer diagnostic methodologies available, testing is simple and less technically demanding. Rapid point-of-care diagnostic tests are particularly important in developing countries, where access to laboratories may be limited and patients are often unable or unwilling to return for test results or treatment [2,3]. Non invasive specimen types are preferable because they overcome some of the barriers associated with the treatment of sexually transmitted infections by being more accessible to the population at risk [4-6]. In addition, it provides the opportunity to diagnose, treat and counsel during the same visit. The Chlamydia Rapid Test®, which was used as the diagnostic tool in this study, is an immunoassay based test that detects chlamydial lipopolysaccharide (LPS). This test uses vaginal swabs as the specimen type and provides a same day result. Performance evaluation indicates that this point of care test can be used for diagnosis of chlamydial infection because of its good sensitivity (83.5%), specificity (98.9%), negative and positive predictive values (98.6% and 86.7% respectively) [2].

Treatment of infection is also effective, inexpensive and readily available and community screening programs have been instituted in some countries to control the progression of disease, prevent the transmission to current or new partner(s), and allow contact tracing, testing and treatment [7].

### Study design and methodology

The aim of this study was to assess the public health burden of genital Chlamydia infection in sexually active females of reproductive age. A prospective cross-sectional study conducted at two hospital sites within Nairobi, Kenya was conducted. The two sites chosen represented a population with different socio-economic status.

**Figure 1:**
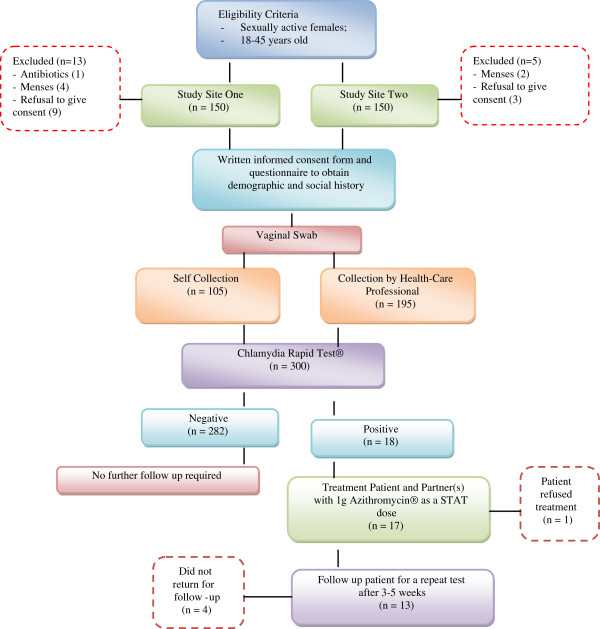
Schematic of the recruitment and testing algorithm.

Adult women aged, 18–45 years, who were currently sexually active and were attending the outpatient clinics and accident and emergency departments at the two study sites during the study period were recruited (Figure 1). Exclusion criteria included women who declined to give informed consent, those who were not accessible for follow-up after testing and those who were on chronic antibiotic treatment. A sample size of 300 was predetermined and calculated using prevalence rates from previous studies conducted in Kenya. Chlamydia testing was conducted on site, either by the principal investigator or a qualified nurse, deemed competent to run the test. The test had an in-built procedural control and known positive and negative control samples (supplied with each kit) were run concurrently with test samples. SPSS (version 15.0) and Microsoft Excel were used for data analysis.

Research and Ethics committees of both participating hospitals (Aga Khan University Research Ethics Committee and St. Mary’s Mission Hospital, Nairobi, Kenya) approved the study.

## Results

Patients were recruited over a period of five months, July to November, 2010. 150 women from each study site (a total 300 women) fulfilled the eligibility criteria (see Figure 1). Women were recruited equally from the different clinics sampled.

Table 1 below highlights the social and demographic differences between the two study populations.

**Table 1:** Summary of social and demographic characteristics of patient population at the two study sites

**Patient characteristic**	**Site one**	**Site two**
**Number of participants**	150	150
**Marital status**	Married	Married
	(95/150, 63.3%)	(110/150, 73.3%)
**Contraceptive use**	No method	No method
	(29%, 43/150)	(52%, 78/150)
**Length of sexual activity**		
**<15 years**	78.7%, 118/150	72.7%, 109/150
**>15 years**	21.3%, 32/150	27.3%, 41/150
**Greater than 2 lifetime Partners**	42.7%, 64/150	16.7%, 25/150
**Asymptomatic patients with Chlamydia infection**	76.9%, 10/13	80%, 4/5
**Acceptability of vaginal swab compared to endocervical swab**	70.1%, 106/150	84.7%, 127/150
**Patient knowledge about Chlamydia infection**	Did not know what *Chlamydia trachomatis* was	Majority reported it as a sexually transmitted infection
	(117/150, 78%)	(90/150, 60%)

The prevalence of infection in the study population was 6% (18/300) (95% Cl 3.31% to 8.69%) [8]. The majority of the women who tested positive were from site one, 8.67% (13/150) (95% CI 4.17% to 13.17%), while 3.33% (5/150) (95% CI 0.46% to 6.2%) were from site two.

Most participants were aged between 30–35 years, 26.3% (79/300). Frequency of genital Chlamydia was highest amongst women aged 25–30 years, 2% (6/300), followed by females aged 35–40 years and 40–45 years, 1.7% (5/300). Confidence intervals comparing participants’ age group and frequency of infection showed a significant higher risk of acquiring infection in patients aged 25–30 years compared to those between 30–35 years (Table 2).

**Table 2:** Confidence intervals comparing age groups of patients and a positive chlamydia test

**Age**	**Number of participants (n=300)**			**Chlamydia test positive (n=18)**			**Frequency and confidence intervals (%)**
	**Site one**	**Site two**	**Total**	**Site one**	**Site two**	**Total**	
**<20 years**	4	2	6	0	0	0	0 (0–0)
**20-25 years**	21	27	48	0	0	0	0 (0–0)
**25-30 years**	39	38	77	4	2	6	33.3 (22.2 – 44.4)
**30-35 years**	40	39	79	2	0	2	11.1 (3.7 – 18.5)
**35-40 years**	26	21	47	3	2	5	27.8 (17.2 – 38.3)
**40-45 years**	20	23	43	4	1	5	27.8 (17.2 – 38.3)

Patients from site one were generally of a higher socioeconomic standing than those from site two; parameters used to assess this difference included patient education, monthly income and rent. More patients, 51% (76/150), from site one had a graduate education compared to 3.3% (5/150) from site two. Only 14% (21/150) of patients from site one had no income compared with 35% (53/150) from site two.

55.6% (10/18) of women with a positive Chlamydia test result were older than 20 years old at first sexual encounter. No significant difference was present when odds ratios were calculated to determine if age at sexual onset (p=0.753), duration of sexual activity (p=0.57) and number of lifetime partners (p=0.928) predisposed to genital Chlamydia infection (Table 1).

77.7% (233/300) of women found the vaginal swab more acceptable than an endocervical swab. Of these, 18% (42/233) preferred collecting the sample themselves.

Patients who tested positive were counselled about the infection and offered treatment for themselves and their partner(s). One patient refused treatment. Four patients (22.2%) did not return for follow-up testing. Of the thirteen patients who returned for a follow-up testing one patient re-tested as positive. However, on repeat testing one month later the test was negative without a second dose of antibiotics.

## Discussion

The overall prevalence of 6% is comparable with previous published reports from Kenya, which showed varied prevalence rates of between 4.2% and 21% [9-11]. Although the target populations in those studies differed from ours, it is interesting that the prevalence rates remain similar indicating that the burden of disease has not decreased in recent times suggesting that relevant intervention strategies are still necessary.

The high prevalence noted among women of greater socioeconomic standing suggests that presence of infection in this group has been largely ignored by previous studies from this region. Our study provides new information and highlights the need to institute active opportunistic screening programs across all socioeconomic groups.

Few studies from Kenya or Africa have examined the prevalence of genital Chlamydia with reference to age unlike in the developed world, where this is the fundamental focus. Investigators have found that the prevalence of Chlamydia infection is highest in younger women especially those under the age of 25 years [12,13]. Although this study was not sufficiently powered to determine the effects of age on prevalence rate, the prevalence rate was highest in women aged 25–30 years, 2%, emphasising that even in our population young women are at higher risk of being infected. Due to ethical concerns, our study population was aged between 18 and 45 years, excluding sexually active teenage girls, which could imply that we might have missed an age group with higher burden of infection.

In our study, 50% (150/300) of the women were older than 20 years of age at the time of first sexual encounter. Studies differ, revealing the disparities in social and sexual behaviour and cultural backgrounds between different countries [13,14]. There was no significant difference between the age of first sexual encounter and infection with Chlamydia. The reason for this might be two-fold; first our study population did not include women younger than 18 years and second our sample size was not large enough to detect a difference in the prevalence of infection with respect to age.

Patients of a higher socioeconomic standing, with a higher prevalence of genital Chlamydia infection, had more than two lifetime partners. Our findings reiterate what other studies have shown and it is postulated that multiple partnerships may increase the likelihood of encountering a sexually transmitted pathogen through the increased probability of choosing a partner with infection, while having new or casual sexual contacts may be related to increased risk because of a reduced familiarity between partners [14,15].

The majority of patients who tested positive for Chlamydia were asymptomatic, highlighting the inadequacy of using syndromic management in such patients. This finding reinforces the need to institute screening programs with the use of low cost and rapid point of care testing to prevent potential spread of infection in susceptible populations.

This is the first study in Kenya using a rapid point-of care diagnostic test with a non invasive vaginal swab as the specimen type enabling patients to get tested and treated within one clinic visit. The majority preferred the non invasive vaginal swab as compared to the conventional endocervical swab for specimen collection.

Rates of contraceptive use were low, possibly due to most women being married and in a monogamous relationship. Few women preferred barrier contraceptives therefore increasing their risk of becoming infected with genital Chlamydia and indeed with other sexually transmitted infections.

Most women had little or no knowledge about genital *Chlamydia trachomatis* as campaigns on sexually transmitted infections in this region tend to focus more on other infectious agents such as HIV, gonorrhoea and herpes simplex. The asymptomatic nature of this infection accentuates the need to educate patients about associated risk factors and available testing modalities.

## Conclusion

Prevalence of genital Chlamydia remains significant in our population especially in women living in highly urbanised areas. This new information provides evidence for the need to implement active opportunistic screening in young sexually active women in our population in patients of all socioeconomic groups. Point of care tests can be employed for infection detection, enabling rapid screening of large numbers with provision of a same day result and treatment if required.

There is a considerable gap in current awareness about genital Chlamydia and an urgent need to prioritise patient and community education, so that young sexually active females are aware of the inherent risk factors that can predispose them to infections.

## Abbreviations

CI: Confidence Interval; STI: Sexually transmitted infection.

## Competing interests

The authors declare that they have no competing interest.

## Authors’ contributions

RK and GR were involved in study conception and design. RK, TO and WPK assisted with data collection and study coordination. RK and WS performed the data analysis. All authors were involved with drafting the manuscript. All authors read and approved the final manuscript.
